# Bioactive Potential of Terpenes from Mediterranean Scrub Plants: A Review

**DOI:** 10.3390/molecules30214268

**Published:** 2025-11-01

**Authors:** Ismael Montero-Fernández, Natividad Chaves Lobón, Laura Nogales Gómez, José Blanco-Salas, Juan Carlos Alías Gallego

**Affiliations:** 1 Department of Analytical Chemistry, Faculty of Science, Universidad de Extremadura, 06006 Badajoz, Spain; 2 Research Institute of Agricultural Resources (INURA), Campus Universitario, Avda. de la Investigación, s/n, 06006 Badajoz, Spain; 3 Department of Plant Biology, Ecology and Earth Sciences, Faculty of Science, Universidad de Extremadura, 06006 Badajoz, Spain; natchalo@unex.es (N.C.L.); lnogalesg@unex.es (L.N.G.); blanco_salas@unex.es (J.B.-S.); jalias@unex.es (J.C.A.G.); 4 Dehesa Research Institute (INDEHESA), Campus Universitario, Avda. de la Investigación, s/n, 06006 Badajoz, Spain

**Keywords:** phytochemistry, terpenes, bioactivity, medicinal plants

## Abstract

The Mediterranean ecosystem is characterized by marked seasonality; it is composed of species such as shrublands that are subjected to high levels of water and thermal stress, making these species an important source of secondary metabolites of significant chemical and ecological interest. In this work, 21 plants were selected from the Mediterranean scrub. These abundant and characteristic representations of the ecosystem produce a total of 197 terpenes. The majority of these are monoterpenes (46.70%), followed by sesquiterpenes (38.07%), with a minority of diterpenes (5.53%) and triterpenes (10.15%). Tetraterpenes accounted for only 0.5% of the total compounds in the species studied, corresponding to only 1%. The major terpenes include 1,8-cineole, terpinen-4-ol, α-terpineol, borneol, camphor, γ-terpinene, limonene, linalool, o-cymene, α-tujene, α-pinene, β-pinene, sabinene, myrcene, β-phellandrene, and β-caryopylene. Species such as *Pistacea terebinthus*, *Rosmarinus officinalis*, *Cistus ladanifer*, *Myrtus communis*, *Lavandula stoecha*, and *Thymus mastichina* contain the most terpenic compounds in their chemical composition. Furthermore, these metabolites are involved in various biological functions, including antimicrobial, antioxidant, neuroprotective, antibacterial, cardiovascular, analgesic, antitumor, and insecticidal activities, among others. Various terpenes present in Mediterranean scrub species, such as 1,8-cineole, α-pinene, limonene, borneol, and terpinen-4-ol, have demonstrated synergistic effects that enhance their antimicrobial, insecticidal, and neuroprotective properties. These interactions between compounds make the natural extracts more effective than they would be individually, increasing their therapeutic and biotechnological value. The synergism among terpenes suggests a promising approach for developing more effective and sustainable phytotherapeutic products.

## 1. Introduction

Mediterranean ecosystems are distributed across various regions of the world, including the Iberian Peninsula [1], and characterized by warm, dry summers and humid, mild winters. Evapotranspiration involves three times the amount of precipitation, causing enormous water stress. These ecosystems have traditionally been considered marginal areas compared to more productive ecosystems. [2]. In the Iberian Peninsula, these ecosystems develop in soils of low fertility, with droughts in summer and frosts in winter [3]. Due to these stressful conditions, plants have developed specific morphological and physiological adaptive mechanisms [4].

Fragile Mediterranean landscapes have been used and transformed since ancient times for agricultural and livestock purposes [5,6]. Also, their species have been used for medicinal purposes by the inhabitants. Thus, medicinal plants have proven to be notable reservoirs of pharmacologically active secondary metabolites. Their relevance also extends to the food, cosmetic, and pharmaceutical industries. Currently, the abandonment of traditional agricultural practices, together with climate change, is altering the structure and dynamics of these ecosystems, with increasing amounts of scrubland and the appearance of invasive species [7]. These circumstances not only place traditional lifestyles based on the exploitation of Mediterranean species at risk, but also limit the potential uses of their bioactive molecules. The growing global demand for terpenes, driven by their application in sectors such as medicine, food, perfumery, and agriculture, has consolidated their commercial interest and potential as a strategic natural resource [8].

Plant molecules are divided into two categories based on their metabolic functions. On the one hand, there are primary metabolites, which participate in basic metabolic functions, and on the other hand, we have secondary metabolites, which perform specific functions in the plant [9]. The main groups of secondary metabolites in plants are terpenes, phenols, and alkaloids. Among the physiological adaptations of Mediterranean scrubland is the production of abundant secondary metabolites, mainly of terpene origin and in the form of essential oil. Essential oils are volatile and hydrophobic compounds produced by plants and composed of simple phenols, monoterpenes, and sesquiterpenes, the latter of which act as phytoalexins and antibiotic compounds produced by plants in response to the appearance of pathogenic microorganisms [10].

## 2. Chemical Structure of Terpenes

**Figure 1:**
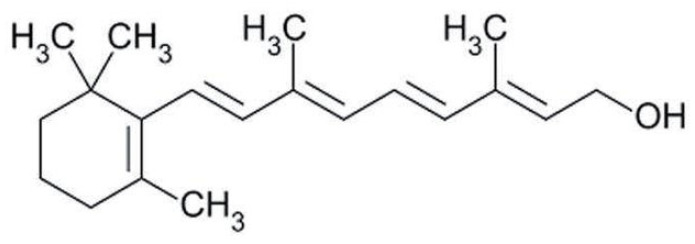
Basic structure of terpenes.

Terpenes are formed from isoprene units (2-methylbuta-1,3-diene) (Figure 1) and its double bond isomer, dimethyl pyrophosphate, and can form linear or cyclic structures through chemical pathways of mevalonate and methylerythriol phosphate [11,12].

The classification of terpenes is carried out based on the number of carbon atoms present in the molecule, the basic unit of which is formed by five carbon atoms, called monoisoprene (C5). These compounds perform a protective function against animals and thermal stress conditions and are classified as monoterpenes (C10), sesquiterpenes (C15), diterpenes (C20), triterpenes (C30), and tetratherpenes (C40) (Table 1) [13].

For the biosynthesis of terpenes, the mevalonate pathway is the only one that can produce precursors in animals and fungi. This occurs from the successive juxtaposition of their precursor molecules, isopentenyl diphosphate (IPP) and its isomer dimethylallyl di-phosphate (DMAPP), in a head-to-tail or tail-to-head fashion, which are produced in the cytosol from mevalonic acid (MVA) or in plastids from pyruvate and 3-phosphoglycerate (MEP) [14,15].

Different types of terpenes have diverse biological properties associated with them [9,16,17]. For example, monoterpenes play an important role in plants due to their antimicrobial, antifungal, and antiviral properties, and these types of compounds usually resist strains of *S. aureus* and *E. coli* [18]. On the other hand, sesquiterpenes help plants grow and present resistance to certain types of microorganisms such as *Bacillus subtilis* and *Staphylococcus aureus* [19]. It is important to highlight other types of terpenoids, such as sesquiterpenes; although uncommon, they present interesting biological activities [20,21,22,23,24].

For these reasons, this work aims to review the terpene composition of the main species composing Mediterranean scrub, as well as their biological potential. Their characterization will enable us to identify the true biotechnological potential of terpene compounds, including their antimicrobial, anti-inflammatory, antioxidant, and neuroprotective activity and potential application in the pharmaceutical, agri-food, and biotechnology sectors. Although studies on individual species exist, an integrative review analyzing the diversity of terpenes in the main plants of the Mediterranean scrubland is still lacking [4,8,17].

## 3. Species Studied

The most representative genera of Mediterranean scrub and trees were identified: *Cistus*, *Cytisus*, *Rosmarinus*, *Lavandula*, *Thymus*, *Erica*, *Pistacea*, *Myrtus*, *Rubus*, and *Calluna*. The *Quercus* genus was added because it is the most predominant and relevant in the tree stratum. In total, 21 species were selected for review. Most of these species are characterized as aromatic, with a high presence of terpenes: *Cistus ladanifer*, *Cistus salvifolius*, *Cistus monspeliensis*, *Cistus albidus*, *Cytisus multiflorus*, *Cytisus scoparius*, *Erica manipuliflora*, *Erica scoparia*, *Calluna vulgaris*, *Myrtus communis*, *Pistacia lentiscus*, *Pistacia terebinthus*, *Rosmarius officinalis*, *Quercus ilex*, *Quercus suber*, *Arbutus unedo*, *Erica australis*, *Lavandula stoechas*, *Thymus mastichina*, *Thymus vulgaris*, and *Rubus ulmifolius.* These Mediterranean plants were selected based on their promising profile of terpene compounds produced by the plant for various functions such as defense, communication, hormone production, and different biological and pharmacological activities [25]. A systematic bibliographic search was conducted on the databases Scopus, ScienceDirect, SciELO, PubMed, Web of Science, and ResearchGate, considering publications from the period 2010–2024. The search strategy combined terms related to the compounds of interest (terpenes, monoterpenes, sesquiterpenes, essential oils, and volatile organic compounds), the ecosystem (Mediterranean, maquis, matorral, garrigue, and shrubland), and the main plant genera characteristics of the Mediterranean scrub (*Cistus*, *Rosmarinus*, *Thymus*, *Lavandula*, among others). Boolean operators (‘AND’ and ‘OR’) and quotation marks for exact phrases were applied to optimize information retrieval. Studies were included if they provided data on the chemical composition of terpenes in Mediterranean species, as well as their biological activity and biotechnological potential. In total, 21 species were selected, based on their ecological abundance, taxonomic representativeness within the Mediterranean scrub ecosystem, and phytochemical relevance due to a high content of secondary metabolites of interest.

## 4. Identified Terpenes

In total, 197 terpene compounds have been identified in Mediterranean scrub plants (Table 2). As can be seen, their presence varies considerably between species. Monoterpenes comprise the majority of compounds, with a total of 92 monoterpene compounds representing 46.70% of the compounds studied. Sesquiterpenes follow with 75 compounds (38.07%). In order of abundance, these are followed by triterpenes, with 20 compounds and 10.15% of the total. Finally, the minority of terpene families found are diterpenes, with only five compounds representing 2.53% of the total, and only one tetraterpene was identified, representing 0.5% of the total.

Analyzing the distribution of compounds by species (Table 3), monoterpenes and sesquiterpenes comprise the majority of compounds in 12 of the species, with 100% of monoterpenic compounds for *Q.i.* The percentage of plants with terpenic compounds decreases from 100% for *Q.i* to 42.85% for *E.a.*, and sesquiterpenic compounds reached the highest percentage for *C.a.*, where all of the identified compounds were sesquitermenes. Values of 59.2 were adopted for the species *C.m.* for low quantities of this family of compounds for *C.sc*., constituting 4.76%. On the other hand, diterpenic compounds were only found in four of the studied species and were found at percentages of 23.56% for *C.mo*, comprising only 2.39% of said compounds for *C.l.* Triperpenes are the main compounds for the species *C.v.*, with 11 compounds found, constituting 78.57% of the total; teraterpenes constituted 21.43% of the total for the same species. They were only identified in three species. Conversely, in *A.u.*, a tetraterpene compound was found constituting 3.59% of the total compounds found in this plant.

As can be seen in Table 2 and Table 3, the presence of these compounds varies between species, resulting in a high qualitative variation. Some compounds were only identified in one species, while others were identified in most species. In this review, we describe the biological and biotechnological functions of these major compounds. Limonene was the most frequently identified compound, found in 13 species, followed by the two isomers of pinene (α-pinene and β-pinene), found in a total of 12 species. Next, borneol was found in 11 species; other terpenes such as 1,8-cineole, terpinen-4-ol, p-cymene, and sabinene appeared in 10 species. α-terpineol and the sesquiterpene caryopylene were found in nine of the species studied. The compounds camphor and α-thujene were found in eight of the species studied; the terpenes myrcene, β-phellandrene, γ-terpinene, and linalool were found in seven of the species studied. We did not consider terpenes present in less than seven species in this work (a third of the species analyzed).

There were variations between species regarding both the type and number of compounds, with large differences in their concentrations. Table 4 contains the variations in concentrations of major terpenes measured as a percentage in two-thirds of the Mediterranean scrub species studied. The most frequent compound among the Mediterranean scrubs studied was limonene; however, this was not the main compound in most of the species where it was present, ranging from trace concentrations in *R.u.* [58] to concentrations of 17.8% in *M.c.* [54,55]. Of the two pinene isomers, α-pinene was found mostly at concentrations of up to 49.65% in *C.l.* [47,48,49,50]. β-pinene was found at lower concentrations compared to α-pinene, with the highest concentration identified in *E.s.* (26.1%) [45]. Borneol, another of the major terpenes, was found at significant concentrations in *P.l.* (50.6%) and concentrations of 4.8% in *A.u.* [38,39,40]. 1,8-cineole was present in eight of the species studied, with varying concentrations from 0.3% for *C.l.* [47,48,49,50] to 33.8% for *R.o*. One of the major compounds found was terpinel-4-ol, at concentrations ranging from 0.2% for *E.s.* to 0.38% for *R.o.* [46] p-cymene was also found at low concentrations in eight of the species studied, with the exception of *P.l.* (22.1%) [56]. Sabinene, found in 11 of the species, presented a higher concentration of 32.7% in *P.l.*, similar to myrcene, the majority of which was found in this plant (16.9%) [57]. α-thujene was found in five of the species at concentrations lower than 1% with the exception of *P.t.* (1.18%) [36,37] and *P.l.* (11.6%) [56]. Other terpenes present in 10 of the species included α-terpinene, varying from 0.06% for *L.s.* [59,60,61,62] to 3.53% in *C.mo.* [32]. Camphor had the highest concentration in *P.l.* (41.6%) [56] and the lowest concentration in *P.t.*, ranging between 0.8 and 3.4% [36,37]. On the other hand, linalool was only found in five of the plants studied and at very low percentages, compared to its higher concentration in *C.sc.* (3.08%) [63,64,65]. Finally, β-phellandrene was found at a higher concentration in *C.a.* (7.48–36.9%) compared to the other Mediterranean plants studied [41,42].

### 4.1. Activity of the Most Common Terpenes Found in Mediterranean Scrub Plants

Most terpenes with common biological activities have antiviral, antibacterial, antifungal, insecticidal, antitumor, anti-inflammatory, neuroprotective, cardioprotective, gastroprotective, and analgesic properties, among others. Table 5 shows the biological activities attributed to each of the most frequently analyzed terpene compounds present in the selected Mediterranean scrub species [65,66,67,68,69,70,71,72,73,74,75,76,77,78,79,80,81,82,83,84,85,86,87,88,89,90,91,92,93,94,95,96,97,98,99,100,101,102,103,104,105,106,107,108,109,110,111,112,113,114,115,116,117,118,119,120,121,122,123,124,125,126,127,128,129,130,131,132,133,134,135,136].

#### 4.1.1. Antimicrobial Activity

One of the main properties of essential oils is their antimicrobial activity, which is being increasingly used in the biotechnology industry. The antimicrobial activity of essential oils from aromatic plants stems from the set of terpenes that make up their chemical composition [137]. The terpene compounds studied in this work all showed antimicrobial activity. Thus, plant extracts with these compounds also have this property. T.v. exhibits antibacterial action against *Gram-positive* and *Gram-negative* bacterial strains [137] as well as other pathogenic bacteria such as *Staphylococcus aureus*, *Bacillus cereus*, *Escherichia coli*, *Proteus vulgaris*, *Proteus mirabilis*, *Salmonella typhi*, *Salmonella typhimurium*, *Klebsiella pneumoniae*, and *Pseudomonas aeruginosa* [138]. The species C.sc. also exhibits antimicrobial activity against large-sized positive bacteria such as *Staphylococcus aureus* and *Bacillus* spp. [139,140]. Terpenic extracts of L.s. undertake activity against *Gram-positive* and *Gram-negative* bacteria, and have susceptibility against *Escherichia coli*, *Pseudomonas aeruginosa*, *Bacillus subtilis*, and *Staphylococcus*. The terpenes with this property present in Mediterranean scrub plants include the following: 1,8-cineole [65,66,67,68,69,70], terpinen-4-ol [75,76,77,78,79,80,81], α-terpineol [42,43,44,45,46,47,48,49,50,51,52,53,54,55,56,57,58,59,60,61,62,63,64,65,66,67,68,69,70,71,72,73,74], borneol [66,82,83,84,85,86], camphor [87,88], χ-terpinene [89,90,91,92], limonene [93,94,95,96,97,98,99], linalool [100,101,102,103,104,105], o-cymene [106,107,108,109,110,111], α-thujene [112,113], α-pinene [114,115,116,117,118,119], sabinene [132,133,134], myrcene [125,126,127,128,129], β-phellandrene [130,131], and β-caryopillene [132,133,134,135,136].

#### 4.1.2. Anti-Inflammatory Activity

One of the main properties of the terpenes present in Table 4 is their anti-inflammatory characteristic. Inflammation is a defense response triggered in a part of the body to various inflammatory agents as a result of an immunological process, whether due to microbial conditions, physicochemical factors, or foreign bodies. In response to this inflammation, species experience high fever and leukocytosis [42,76]. The anti-inflammatory response of terpenoids originates from the inhibition of the nuclear transcription factor-kB (NF-kB) and mitogen-activated protein kinase (MAPK) signaling pathways [138,139].

In addition, they modulate the generation of cytokines, chemokines, and molecular cell adhesion, while targeting cell signaling pathways [141]. Among the plants included in the Mediterranean scrub with these types of properties, *E.s.* stands out, traditionally used for bladder inflammation, kidney disorders, and urinary tract inflammation [142]. On the other hand, C.l. has anti-inflammatory properties and a healing action [143]. A complete list of compounds identified in Table 4 with these properties is 1,8-cineole, terpinen-4-ol, α-terpineol, camphor, γ-pinene, linalool, o-cymene, β-pinene, and myrcene.

#### 4.1.3. Neuroprotective Activity

Some of the compounds studied are attributed with a neuroprotective function. One of these is terpinen-4-ol, which, due to its antioxidant capacity, is capable of eliminating free radicals and reactive oxygen species, in addition to forming chelates with metal ions to reduce the effects of oxidative stress on the nervous system, among others [144]. Among the neuroprotective activities of this compound, enzymes such as acetylcholinesterase, butylhydroxycholinesterase, and urease are inhibited and involved in the synthesis of neurotransmitters [145]. For their part, α-pinene and 1,8-cinelol act as regulators of gene expression, protecting PC12 cells against apoptosis induced by oxidative stress in nervous system cells [146]. α-pinene acts as a protector against apoptotic cell death and brain diseases [147]. Another terpene with neuroprotective action is borneol, which has anti-amyloid effects, both in SH-SY5Y cells and motor neurons [148]. p-cymene also has neuroprotective effects due to its cholinergic effect when regulating gene expression in *Caenorhabditis elegans* [149]. Limonene, among its biological properties, also has a neuroprotective role in neurodegenerative diseases such as Alzheimer’s, multiple sclerosis, epilepsy, anxiety, and stroke [150]. In addition, limonene induces neurotoxicity against Aβ42 in droscophila, a model developed in the fruit fly [151]. Finally, another compound that has neurotoxic activity is myrcene [130]. This leads us to propose that the species *P. lentiscus*, *L. stoechas*, and *T. vulgaris* are reservoirs of compounds involved in neuroprotective function [152].

#### 4.1.4. Gastroprotective Activity

Another therapeutic application of terpenes is their gastroprotective function against gastric ulcers, a pathology that affects more than 14.5 million people worldwide [152]. In addition, they have been shown to be effective against one of the most important gastrointestinal pathogens, the *Gram-negative* bacterium *Helicobacter pylori* (*H. pylori*), which infects almost 4.4 billion people worldwide [125,153,154]. They also reduce the adverse effects caused by the prolonged use of certain medications, such as stomach cancer [155]. There is an urgent need to search for new and effective but non-toxic, easily accessible, and affordable medications [155]. One compound that has this potential is α-terpineol, which is present in the essential oils of *C.s.*, *A.u.*, *E.a.s* and *L.s.*. α-*terpineol* and stands out for its antispasmodic, contraceptive, and immunostimulant properties [82]. Because this compound is common in flowers, herbs, leaves, and fruits, its biomolecule is easy to extract. Its only limitation is that it is a volatile compound, insoluble in water, and has a short life in the bloodstream of only 12.5 min [156]. Therefore, to improve its solubility, reduce its volatility, and enhance bioavailability, encapsulation techniques are used to ensure that it reaches the digestive tract with a controlled and prolonged release to its target region [157,158].

#### 4.1.5. Cardioprotective Activity

Cardioprotection involves all mechanisms that contribute to preventing or correcting myocardial damage [75,159,160,161,162,163]. The main compounds listed in Table 4 associated with this property are terpinen-4-ol, 1,8-cineole, borneol, β-pinene, and β-caryopylene. These compounds are present in plants such as *C.s.*, *C.m.*, *P.t.*, *E.s.*, *R.o.*, *C.l.*, *C.m.,* and *L.s.*. Terpinel-4-ol has been studied both individually and as a constituent of essential oils, and has been used in cardiovascular treatment in animals with arterial hypertension. It results in a significant expansion of the blood in addition to improving muscular relaxation of the blood vessels. This compound can also act as an antioxidant by decreasing LPO levels and reducing metal ions such as Ca^2+^ and Fe^3+^ while mitigating the effects of free radicals, and increasing the levels of glutathione transferase and superoxide dismutase activity [76,145]. Borneol also shows cardioprotective activity [85,87]. Borneol, a highly lipid-soluble bicyclic terpenoid, is widely used in oriental medicine due to its various therapeutic actions, including, among other cardioprotective properties, the alleviation of acute heart diseases [66]. In addition, it suppresses inflammatory responses by downregulating the mRNA expression of interleukin-1β and interleukin-6. It also promotes angiogenesis by upregulating hypoxia-induced factor 1α and CD34, which exerts positive cardiovascular effects and alleviates pathological damage [163].

#### 4.1.6. Insecticidal Activity

Commercial formulations of terpene-based pesticides are currently being used [164,165,166,167,168]. In particular, α, β-unsaturated terpene ketones, which are produced naturally by some plants, have a potent fumigant effect and toxicity against certain insect pests [169]. Essential oils can be used as an alternative to synthetic pesticides due to their low toxicity and high structural diversity. The main terpenes found in Mediterranean scrub plants with insecticidal properties are 1,8-cineole, α-terpineol, limonene, o-cymene, and α-pinene. There is evidence that not all terpenes have the same insecticidal power, with monoterpenes containing carbonyl groups that are more toxic than molecules containing alcohols or hydrocarbons [170,171,172]. Among the plants studied, *C.m.*, *E.a.*, *R.o.*, *C.l.*, *C.m.*, and *L.s.* stand out with greater insecticidal potential. Essential oils with a high presence of limonene are toxic to numerous insect pests [171,172,173]. α-pinene also has insecticidal properties in synergism with other terpenes such as β-pinene, sabinene, and γ-terpinene, against Musca domestica larvae [90,93,114,115,133,134].

#### 4.1.7. Synergistic Interactions Between Terpenes

In addition to their individual biological activity, several studies highlight that many terpenes present in Mediterranean scrub species can act synergistically, enhancing their therapeutic and ecological efficacy. This synergy refers to the interaction between two or more compounds that, when combined, generate an effect greater than the sum of their individual effects. In the case of the compounds identified in Table 4, it has been observed that multiple combinations can present enhanced effects on various biological activities, especially in antimicrobial, anti-inflammatory, insecticidal, and neuroprotective contexts. The combination of 1,8-cineole with terpinen-4-ol enhances its antimicrobial capacity, complementing the destabilization of cell membranes and the inhibition of bacterial enzymatic activity [9]. Similarly, α-terpineol and linalool, which are both present in several species of the Mediterranean scrub, have synergy in their antioxidant and antimicrobial properties, improving efficacy against pathogens by combining bacteriostatic and bactericidal properties [18].

In the case of insecticidal activity, limonene, γ-terpinene, α-pinene, and sabinene have synergistic effects when used in mixtures, increasing toxicity against pest insects such as Musca domestica [173]. This interaction improves the ability of the compound to penetrate the insect and enhances its neurotoxic action. In the field of neuroprotection, the combination of borneol and linalool exerts beneficial effects by reducing neuronal oxidative stress and modulating the activity of enzymes such as acetylcholinesterase, with implications for neurodegenerative diseases such as Alzheimer’s [149]. Likewise, α-pinene, in the presence of other monoterpenes such as limonene and sabinene, can act as a cofactor in the protection of cells in the nervous system [145,151]. On the other hand, it has been suggested that β-caryophyllene, together with linalool or limonene, enhances anti-inflammatory potential by acting on different cellular signaling pathways, including NF-κB and MAPK, suggesting a broader therapeutic use through terpene mixtures [16].

From an ecological perspective, this chemical synergy could represent an adaptive strategy for Mediterranean plants, enhancing their defense against extreme conditions and pathogens through specific combinations of terpenes with complementary or enhanced activity. This metabolic cooperation allows for more effective responses to different types of biotic or abiotic stress, enhancing the biotechnological potential of these species. From all this, it can be concluded that Mediterranean scrub plant extracts should not be considered solely for the presence of individual compounds, but rather for the complete terpene profile and the interactions between them, which may be decisive for their application in phytotherapy, pharmacological formulations, natural cosmetics, and biological control.

## 5. Conclusions

In this review, phytochemical information was compiled and analyzed for representative species of the Mediterranean scrubland, revealing their richness in terpene compounds with relevant biological activities, such as carvacrol, 1,8-cineole, borneol, and thymol. These volatile profiles not only reflect the pharmacological and cosmetic potential of these plants but also suggest potential synergistic effects between terpenes that may enhance their efficacy. Revaluing these species as a source of bioactive molecules not only has a scientific and ecological impact but also an economic one, opening up opportunities for the development of natural products with added value. This perspective can promote the sustainable use of Mediterranean biodiversity, especially in rural areas, contributing to the revitalization of local economies through their integration into sectors such as phytotherapy, agroindustry, and natural cosmetics.

## Figures and Tables

**Table 1:** Classification of terpenes.

Isoprene Units	Number of Carbons	Terpene Nome
2	10	monoterpene
3	15	sesquiterpene
4	20	diterpene
6	30	triterpene
8	40	tetraterpene
<8	>40	politerpene

**Table 2:** Terpenes found in the Mediterranean scrub plants studied.

Terpene Family	*T.v.*	*C.s.*	*C.m.*	*Q.s.*	*Q.i.*	*P.t.*	*A.u.*	*C.a.*	*E.m.*	*E.a.*	*E.s*	*S.r.*	*C.l.*	*C.mo.*	*C.sc.*	*C.v.*	*M.c.*	*P.l.*	*R.u.*	*L.s.*	*T.m.*
MONOTERPENES																					
β-linalool	+	+				+								+			+				
1,8-cineole	+		+		+						+	+	+		+		+		+	+	
Terpinen-4-ol	+	+	+			+					+	+	+	+			+				+
α-phellandrene	+				+							+	+		+		+	+			
α-terpineol	+	+			+		+			+			+		+		+	+		+	
borneol	+	+	+			+					+	+	+		+		+			+	+
camphor	+					+					+	+	+		+			+		+	+
carvacrol	+										+	+		+	+						
citronellol	+										+	+			+						
eucalyptol													+	+							
eugenol											+	+		+	+						
γ-terpinene						+					+	+	+	+	+					+	
geraniol	+	+									+			+			+				+
limonene	+	+			+	+					+	+	+		+		+	+	+	+	+
linalool					+		+				+	+	+		+				+	+	
menthol	+	+									+				+						
*o*-cymene	+	+			+						+		+	+			+	+	+	+	+
*p*-menth-1-en-9-al														+							
pulegone											+				+						
terpinen-4-ol	+											+			+		+			+	+
verbenene						+							+		+					+	
Isobutyl isobutyrate																	+				
α-thujene	+	+	+		+						+						+			+	+
α-pinene	+	+	+		+	+					+	+	+				+	+		+	+
β-pinene	+	+	+			+			+	+	+		+				+	+		+	+
β-myrcene						+			+		+		+				+				
3-carene		+							+								+			+	
*€*-β-ocimene						+											+			+	
cryptone																	+				
myrtenol		+					+				+		+				+			+	
Bicyclogermacrene																	+				
*p*-menth-1,5-dien-8-ol																	+				
α-campholenal																	+			+	
cis-menth-2-en-1-ol																	+				
α-terpinene		+									+	+	+				+			+	
*Z*--ocimene						+			+								+			+	
Sabinene	+				+	+			+		+		+				+	+		+	+
myrcene	+				+							+						+	+	+	+
β-phellandrene	+				+	+					+		+					+		+	
δ-terpinene						+												+			+
perillene																			+		
*p*-cymene													+							+	
a-fenchene	+										+	+								+	
α-pinocarveol													+							+	
*cis*-verbenol																				+	
pinocarvone													+							+	
*cis*-pinocamphone																				+	
*p*-cymen-8-ol												+								+	
*t*-caveol																				+	
carvone	+	+									+									+	
thymol		+									+	+								+	
tricylene	+				+							+	+							+	
hortrienol																				+	
nerol	+	+																			+
isopinocamphone													+								
3-carene												+									
E-pinocarveol												+									
β-terpinene	+										+										
3-carvene	+										+										
4-carvene											+										
isopulegol											+										
estragol											+										
germanene						+				+	+										
Anethole											+										
Menthone											+										
2-*trans*-hexenal										+											
*n*-octanal										+											
*n*-nonanal							+			+											
*n*-decenal	+						+														
E-2-nonenal							+														
β-cyclocitral							+														
octanol							+														
(*E*,*E*)-2,4-heptadienal							+														
(*E*,*Z*)-2,6-nonadienal							+														
*E*-2-decenal							+														
(*E*,*E*)-2,4-nonadienal							+														
(*E*,*Z*)-2,4-decadienal							+														
-piperitrol						+															
*E*-epoxycimene						+															
β-cuberene						+															
3-carene						+															
2-carene						+															
safranal			+																		
α-cadiol		+	+																		
β-cadiol			+																		
*o*-guayacool		+																			
*p*-mentha-1(7),8-diene	+																				
*t*-pinocarveol																	+				
terpinolene											+										
*p*-menth-1,4-dien-8-ol														+	+					+	
camphene													+				+			+	
platanic acid							+														
SESQUITERPENES																					
myrtenyl acetate																	+			+	
α-terpinyl acetate	+																+			+	
Neryl acetate		+									+		+				+				
Geranyl acetate	+									+							+			+	
Methyl eugenol																	+				
*E*-β-caryophyllene	+										+						+				
α-humulene		+				+						+	+				+				+
valencene																				+	
γ-gurjunene																				+	+
isoledene																				+	
α-ylangene													+								
α-cubebene		+										+									
thymol acetate												+									
neroidol											+										
carvacol acetate	+										+										
α-farnese										+											
*E*-2-undecenal							+														
β-ionone			+																		
Spathulenol			+																		
β-gurjunene			+																		
(*Z*)-*t*-bergamotol			+																		
*E*-β-damascone			+																		
*E*-2-hexylcinemaldehide			+																		
isopulegyl acetate		+																			
trans-α-ambrinol		+																			
*cis*-α-ambrinol		+																			
*cis*-muurole-4(14)5-diene		+																			
allo-aromadendrene		+																			
cadina-1,4-dione		+																			
β-elemene		+																			
β-copaene		+																			
α-caryophyllene						+			+												
germacrene																	+	+		+	
allo-aromadendrene													+							+	+
〈-humulene														+						+	
α-selinene																				+	
γ-muurolene													+								+
viridiflorene		+											+								+
α-muurolene								+				+	+	+							+
β-bisabolene																					+
δ-cadiene								+													+
palustrol													+								
Spathulenol		+											+								
ledol			+					+					+								
β-eudesmol													+								
cadalene		+											+								
chavicol											+										
α-bisabolol			+					+			+										
nerolidol											+										
*cis*-bourbonene										+											
selin-11-en-4-ol								+													
β-bourbonene								+													
α-curcumene								+													
elemol		+						+													
α-zingiberene								+													
α-calacorene			+																		
α-muurolol			+																		
α-lonone			+																		
bornyl acetate			+			+						+	+							+	
lavandulyl acetate	+																			+	
α-copaene		+	+										+							+	
longifolene																				+	
β-caryophyllene	+	+	+				+	+	+			+								+	+
linaly acetate											+										+
α-gurjunene																					+
δ-cadiene						+					+		+					+			
*E*-β-lonone			+																		
globulol		+																			
guaiol		+																			
DITERPENES																					
gibberellic acid														+							
Retinal														+							
Retinol														+							
16-kaurene													+	+							
kaur-16-en			+																		
vulgarol		+	+																		
TRITERPENES																					
α-amyrin							+									+					
α-amyrenone																+					
β-amyrin							+									+					
botulim				+			+									+					
cycloartanol																+					
erythrodiol																+					
friedelin				+												+					
4-epi-friedelin																+					
Lupeol							+									+					
taraxasterol																+					
olean-12-en-3b-23-diol							+														
7-β-hydroxystigmast-4-en-3-one							+														
pomalic acid 3-acetate							+														
β-sitosterol				+			+														
moretenol							+														
9,19-Cyelolamastan-3-ol							+														
lupenone							+														
norolean-12-en							+														
betulinic acid				+																	
sitost-4-en-3-one				+																	
ursolic acids																+					
Sitostanol																+					
Stigmasterol																+					
methylenecycloartanol							+									+					
References	[26,27,28,29]	[30,31]	[32]	[33,34]	[35]	[36,37]	[38,39,40]	[41,42]	[43]	[44]	[45]	[46]	[41,47,48,49,50]	[50,51]	[31,52]	[52,53]	[54,55]	[56]	[57]	[58,59,60,61]	[62,63]

Legend: *T.v.* = *Thymus vulgaris*; *C.s.* = *Cistus salvifolius*; *C.m.* = *Cytsus multiflorus*; *Q.s.* = *Quercus suber*; *Q.i.* = *Quercus ilex*; *P.t.* = *Pistacea terebinthus*; *A.u.* = *Arbutus unedo*; *C.a.* = *Cistus albidus*; *E.m.* = *Erica manipuliflora*; *E.a.* = *Erica australis*; *E.s.* = *Erica scoparia*; *S.r.* = *Salvia rosmarinus*; *C.l.* = *Cistus ladanifer*; *C.mo.* = *Cistus monspeliensis*; *C.sc.* = *Cytisus scoparius*; *C.v.* = *Calluna vulgaris*; *M.c.* = *Myrtus communis*; *P.l.* = *Pistacea lentiscus*; *R.u.* = *Rubus ulmifolis*; *L.s.* = *Lavandula stoechas*; *T.m.* = *Thymus mastichina*; + = the metabolite is present in a particular plant.

**Table 3:** Number of terpenes per group and percentage of the total for each species.

	Monoterpenes	%	Sesquiterpenes	%	Diterpenes	%	Triterpenes	%	Tetraterpenes	%
*Q.i.*	11	100								
*P.t.*	21	87.5	3	12.50						
*E.m.*	6	85.7	1	14.30						
*P.l.*	11	84.60	2	15.40						
*R.u.*	5	83	1	17						
*E.s.*	34	82.93	7	15.7						
*T.v.*	27	81	6	19						
*C.sc.*	16	76.10	1	4.76	4	19.14				
*S.r.*	20	71.43	8	28.57						
*M.c.*	28	70	12	30.0						
*C.s*	20	60.0	13	40						
*C.m* *o.*	10	58.8	3	17.64	4	23.56				
*C.l.*	24	57.14	17	40.47	1	3.29				
*A.u.*	13	46.42	2	7.14			12	42.85	1	3.59
*T.m.*	10	45	12	55						
*E.a.*	6	42.85	8	57.2						
*C.m.*	9	30	16	59.2	2	10.80				
*L.s.*	34	69.40	15	30.60						
*C.a.*			11	100						
*Q.s.*							5	100		
*C.v.*							11	78.57	3	21.43

Legend: *Q.i.* = *Quercus ilex*; *P.t.* = *Pistacea terebinthus*; *E.m.* = *Erica manipuliflora*; *P.l.* = *Pistacea lentiscus*; *R.u.* = *Rubus ulmifolis*; *E.s.* = *Erica scoparia*; *T.v.* = *Thymus vulgaris*; *C.sc.* =*Cytisus scoparius*; *S.r.* = *Salvia rosmarinus*; *M.c.* = *Myrtus communis*; *C.s.* = *Cistus salvifolius*; *C.mo.* =*Cistus monspeliensis*; *C.l.* = *Cistus ladanifer*; *A.u.* = *Arbutus unedo*; *T.m.* = *Thymus mastichina*; *E.a.* = *Erica australis*; *C.m.* = *Cytsus multiflorus*; *L.s.* = *Lavandula stoechas*; *C.a.* = *Cistus albidus*; *Q.s.* = *Quercus suber*; *C.v.* = *Calluna vulgaris*.

**Table 4:** Concentration of the majority terpenes expressed as a percentage identified in the different species.

Terpene (%)	*T.v.*	*C.s.*	*C.m.*	*P.t.*	*A.u.*	*C.a.*	*E.m.*	*E.a.*	*E.s.*	*S.r.*	*C.l.*	*C.mo.*	*C.sc.*	*M.c.*	*P.l.*	*R.u.*	*L.s.*	*T.m.*
1,8-cineole	trazes-6.7		0.4						0.5	33.8	0.3		*N.q*.	25.7		0.4	0.22–16.3	
Terpinen-4-ol	0,06	trazes-0.7	0.7	0.26–0.30					0.2	0.38	1.2–1.82	0.46		10.1				0.1
α-terpineol	trazes-2.69	2,6			4.8			0.7					0.62–1.23	0.9	50.6		0.03	
borneol	0.4	0.4–6.3	6.3	0.8					*N.q*.	9.17	0.38–11.1		*N.q*.	0.1			0.8–1.59	trazes-0.9
camphor	0.4–1.79			0.8–3.4					*N.q*.	5.07	11.22		*N.q*.		41.6		1.3–2.8	7.2
γ-terpinene	trazes-30.9			0.25–3.5					*N.q*.	0.65	0.79–1.70	3.53	1.17				0.06–0.2	
Limonene	0.05–7.14	0.1–1.67		1.3–13.5					10.7	3.19	2.8–3.3		*N.q*.	4.1–17.8	4.4	trazes	0.2–1.1	1.07–71.82
Linalool	5.1–76.6				1.2				*N.q*.	0.89			3.08			0.3	trazes-0.41	
p-cymene	trazes	0.4–0.96							3.4		5.42	0.3		0.9	22.1	0.4	0.1	0.4–9.7
α-thujene	0.5–2.84	0.6	0.6						*N.q*.					0.4			0.01	0.1–0.2
α-pinene	0.47–5.7	0.86	0.6	0.8–45.36					6.8	1.86	2.7–49.65			14.7	34.7		0.4–2.96	2.3–7.2
β-pinene	trazes-0.92	2.91	0.7	2.2–20.74			*N.q*.	trazes	26.1		0.61–5.61			4.6	18.3		trazes-1.3	1.72–5.7
Sabinene	trazes-0.2			5.61			*N.q*.		1.3		0.37–16.03			3.1	32.7		0.03–0.1	1.6–3.17
Myrcene	0.09–3.45									1.11	2.71				16.9	0.5	trazes-0.01	2.90–9.81
β-phellandrene	0.21–0.3			1.18					0.3		0.61				11.6		0.09–0.1	
β-caryopillene	2.27–2.68	0.7	1.65–2.40		0.7	7.48–36.9	*N.q*.			0.9							0.03	0.1
References	[26,27,28,29]	[30,31]	[32]	[33,34]	[35]	[36,37]	[38,39,40]	[41,42]	[43]	[44]	[45]	[46]	[47,48,49,50]	[41,50,51]	[31,51]	[52,53]	[54,55]	[56]

Legend: *T.v.* = *Thymus vulgaris*; *C.s.* = *Cistus salvifolius*; *C.m.* = *Cytsus multiflorus*; *P.t.* = *Pistacea terebinthus*; *A.u.* = *Arbutus unedo*; *C.a.* = *Cistus albidus*; *E.m.* = *Erica manipuliflora*; *E.a.* = *Erica australis*; *E.s.* = *Erica scoparia*; *S.r.* = *Salvia rosmarinus*; *C.l.* = *Cistus ladanifer*; *C.mo.* = Cistus mospeliensis; *C.sc. = Cytisus scoparius*; *M.c.* = *Myrtus communis*; *P.l.* = *Pistacea lentiscus*; *R.u.* = *Rubus ulmifolis*; *L.s.* = *Lavandula stoechas*; *T.m.* = *Thymus mastichina*; trazes = concnetration minor of 1000 mg L^−1^; *N.q.* = not detected.

**Table 5:** Functions of the major terpenes found in this work.

Compound	Function	References
1,8-cineole (eucalyptol) 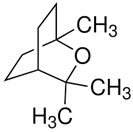	Antihypertensive. cardioprotective. Antioxidant, bronchodilator, analgesic, proapoptotic against airway-related cells, antibacterial (preservative), antinutritional, insecticidal, antifungal effects.	[65,66,67,68,69,70]
Terpinen-4-ol 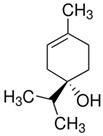	Involved in diseases related to oxidative stress, neurodegenerative disorders, anticancer, cardiovascular diseases, diabetes, insecticide (against *Sitophilus* zeamis and *A. solani*), arrhythmagenic activity in cardiac tissue. Antimicrobial	[75,76,77,78,79,80,81]
α-terpineol 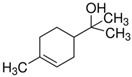	Insecticide (entomopathogenic nematodes), gastroprotective activity, antibacterial, antioxidant, antifungal	[41,71,72,73,74]
Borneol 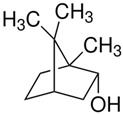	Skin repair activity (inhibition of tyrosinase activity), antibacterial, anti-inflammatory, aardio and cerebrovascular function, antitumor, antifungal analgesic, antipyretic	[66,82,83,84,85,86]
Camphor 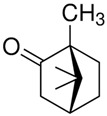	Membrane stabilization. used in the treatment of cognitive deficits anti-inflammatory and antifungal	[87,88]
γ-terpinene 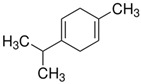	Antitumor, antibacterial, antifungal, biofuel and antioxidant Anti-inflammatory	[89,90,91,92]
Limonene 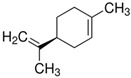	Insecticide, antidepressant, antioxidant, antimicrobial, anti-inflammatory antifungal, anticancer	[93,94,95,96,97,98,99]
Linalool 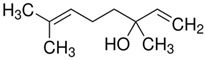	Antibacterial, antinociceptive, antimicrobial, antioxidant, anti-inflammatory, analgesic, anxiolytic, antihyperlipidemics, neuroprotective and antidepressant	[100,101,102,103,104,105]
*o*-cymene 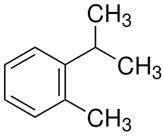	Antioxidant, anti-inflammatory, antiparasitic, antidiuretic, antiviral, antitumor, antibacterial, antifungal, cytoprotective, insecticide and chemotherapeutic Potential	[106,107,108,109,110,111]
α-thujene 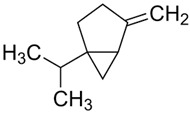	Neurotoxic, Antimutagenic, Immunomodulator, Antidiabetic and antimicrobial	[112,113]
α-pinene 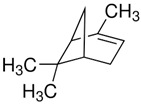	Anti-inflammatory, antioxidant, neuroprotective, biofuel. Hypoglycemic and hypolopidemic activity, Flavoring and flavoring, Antibacterial, Insecticide and herbicide	[114,115,116,117,118,119]
β-pinene 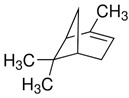	Polymer synthesis, anticoagulant, antibiotic resistance, antitumor, antimalarial, antioxidant, anti-inflammatory, anti-leishmania.	[122,123,124]
Sabinene 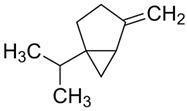	Antimicrobial jet fuel Flavoring	[132,133,134]
Myrcene 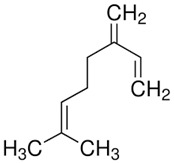	Food additive, anxiolytic, antioxidant, anti-inflammatory, analgesic, antimicrobial, neuroprotective	[125,126,127,128,129]
β-phellandrene 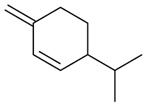	Antifungal, antibacterial	[130,131]
β-caryopillene 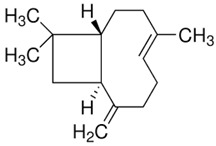	Anti-inflammatory, neuroprotective, flavoring, flavor enhancer, cardioprotective, hepatoprotective, nephroprotector, antioxidant and antitumor	[132,133,134,135,136]

## Data Availability

Not applicable.
